# Protective Performance of Helmets and Goggles in Mitigating Brain Biomechanical Response to Primary Blast Exposure

**DOI:** 10.1007/s10439-022-02936-x

**Published:** 2022-03-16

**Authors:** Xiancheng Yu, Mazdak Ghajari

**Affiliations:** 1 Dyson School of Design Engineering, Imperial College London, South Kensington, London, SW72AZ UK; 2 Centre for Blast Injury Studies, Imperial College London, South Kensington, London, SW72AZ UK

**Keywords:** Blast brain injury, CSF cavitation, Intracranial pressure, Combat helmet, Goggles

## Abstract

The current combat helmets are primarily designed to mitigate blunt impacts and ballistic loadings. Their protection against primary blast wave is not well studied. In this paper, we comprehensively assessed the protective capabilities of the advanced combat helmet and goggles against blast waves with different intensity and directions. Using a high-fidelity human head model, we compared the intracranial pressure (ICP), cerebrospinal fluid (CSF) cavitation, and brain strain and strain rate predicted from bare head, helmet-head and helmet-goggles-head simulations. The helmet was found to be effective in mitigating the positive ICP (24–57%) and strain rate (5–34%) in all blast scenarios. Goggles were found to be effective in mitigating the positive ICP in frontal (6–16%) and lateral (5–7%) blast exposures. However, the helmet and goggles had minimal effects on mitigating CSF cavitation and even increased brain strain. Further investigation showed that wearing a helmet leads to higher risk of cavitation. In addition, their presence increased the head kinetic energy, leading to larger strains in the brain. Our findings can improve our understanding of the protective effects of helmets and goggles and guide the design of helmet pads to mitigate brain responses to blast.

## Introduction

Traumatic brain injury (TBI) is a major cause of death and disability around the world. Many of the TBI cases in military field are caused by the exposures to blasts, mostly resulting from the detonations of Improvised Explosive Devices (IEDs).^27^ These TBI cases are therefore classified as blast induced TBI (bTBI). Military personnel are not the only victims of blasts. According to Action on Armed Violence (AOAV),^1^ explosive weapons caused nearly 30,000 deaths and injuries worldwide in 2019, of which 66% were civilians.

Despite the prevalence of bTBI, its protection is not well studied, particularly the protection against primary blast wave. Modern combat helmets and other protective equipment, such as goggles, are primarily designed to resist ballistic loadings, e.g. shrapnel, bullets and fragments from gun shots or explosions.^8^ However, their protective capabilities against primary blast wave are not well studied. One study found that the blast wave mitigation of modern combat helmet is similar to the 100-year-old historical combat helmets.^26^ This indicates that combat helmets’ protection against primary blast wave may not have been improved over the last 100 years.

The limited research on protection against primary blast wave may be due to the lack of understanding of the mechanisms of primary bTBI. A few experimental studies have shown a link between blast overpressure and brain injury,^7,10,22^ which confirms that primary blast wave alone is able to cause brain injury at different severity. The biomechanical responses of brain to primary blast wave include elevated intracranial pressure (ICP), strain, strain rate, cerebrospinal fluid (CSF) cavitation, etc. It remains unclear which of these biomechanical responses causes brain injury. ICP, strain and strain rate are the commonly used criteria in brain injury assessment.^12,36,37,50^ CSF cavitation is another possible biomechanical response that has also attracted much attention.^6,27,32,40^ Cavitation is a physical phenomenon in which vapour bubbles form in a fluid due to low ambient pressure. The collapse of the bubbles can generate local shock waves and micro-jets, which can damage nearby structures. A previous study analysed post-mortem human brain tissues from both blast and non-blast TBI cases.^35^ Their results showed distinct patterns of brain damage (astroglial scarring) located at the brain tissue-CSF interface, which existed in bTBI cases only. CSF cavitation may provide an explanation for such interface damage. In our recent studies, we used both computational and experimental approaches to investigate the CSF cavitation under blast exposures.^46,47,49^ With a simplified physical head surrogate, we showed that non-lethal blasts can already induce CSF cavitation and the following micro-jets formation at the contrecoup region. We further proposed a mechanism for this phenomenon: the pressure waves transmitting through the skull and tissue simulants are responsible for the generation and collapse of the cavitation bubbles, respectively.^49^

Most previous studies used the ICP, strain or strain rate as criteria to assess the bTBI.^12,18,20,36,37,39,43,50^ Up to date, no studies have investigated the effect of protective equipment on CSF cavitation. Another limitation of the previous studies is the lack of detailed brain anatomy description in the human head model, which may affect the biomechanical responses of brain to blasts. In addition, research on goggles’ performance on mitigating brain’s biomechanical responses is limited.

In this study, our aim is to comprehensively study the protective performance of combat helmets and goggles against primary blast wave exposure. Detailed finite element (FE) models of a combat helmet and goggles were firstly developed. Then, the helmet and goggles models were fitted onto a high-fidelity human head model. We validated the models by comparing the computational results with previous experimental data. Next, the head model was exposed to blast loadings with different intensities and different directions in three configurations: bare head, helmet-head and helmet-goggles-head. We finally assessed the protective capability of helmet and goggles on ICP, CSF cavitation, brain strain and strain rate.

## Materials and Methods

**Figure 1:**
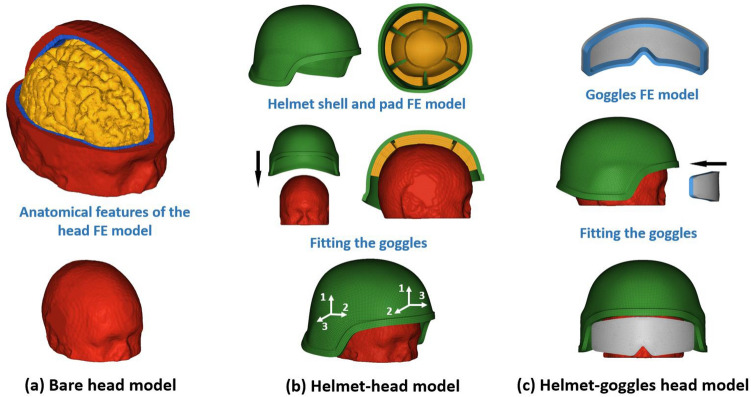
(a) The bare head model. Part of the skin and skull were masked to show the internal anatomical features. (b) The helmet-head model. Preliminary simulation is conducted for helmet fitting. The two coordinate system shows the different orthotropic directions at different locations, where axis 1 and 2 are the two in-plane directions and axis 3 represents the thickness direction. (c) The helmet-goggles head model. Preliminary simulation is conducted for goggles fitting.

We developed three models to investigate the protective performance of a combat helmet and goggles: bare head model, helmet-head model and a helmet-goggles-head model (Fig. 1). The development of the FE models and simulations were conducted using the LS-Dyna nonlinear hydro-code.^21^

### The High-fidelity Human Head Model

A human head model developed in a previous study was used here.^12^ The model includes detailed anatomy of various tissues, including skin, skull, grey and white matter, CSF, ventricles, falx and tentorium (Fig. 1a). The head model consists of around one million hexahedral solid elements and a quarter of a million shell elements, with an average element size of 1.5mm. The material models and properties of key tissues (Table 1) are from Ref. 27 and have been used in our previous work.^47^ The deviatoric response of the scalp, skull and brain were modelled with linear viscoelastic model while their volumetric responses were modelled by defining a bulk modulus. The deviatoric and volumetric responses of the CSF were modelled with a dynamic viscosity and Gruneisen equation of state (EOS), respectively (Table 1). The Gruneisen EOS has been suggested for modelling the volumetric response of CSF in previous studies.^11,14,51^ In our recent study, we used this CSF material model and found good agreement between the predicted contrecoup CSF pressure and the pressure measured in the experiments.^49^

**Table 1:** Material properties of tissues.^27^

Materials	Bulk properties	Shear properties	
Scalp	*ρ*= 1130 kg/m^3^ *K* = 2190 MPa	G_1_ = 355 kPa G_2_ = 399 kPa G_3_ = 35.6 kPa G_∞_ = 408 kPa	*β*_1_ = 0.005 ms^−1^ *β*_2_ = 0.05 ms^−1^ *β*_3_ = 0.5 ms^−1^
Skull	*ρ* = 2000 kg/m^3^ *K* = 10227 MPa	G_1_ = 2289.6 kPa G_2_ = 4708.5 kPa G_∞_ = 4720.3 kPa	*β*_1_ = 0.03 ms^−1^ *β*_2_ = 275 ms^−1^
Brain	*ρ* = 1060 kg/m^3^ *K* = 2189 MPa	*G*_1_ = 50 kPa *G*_2_ = 6.215 kPa *G*_3_ = 2.496 kPa *G*_4_ = 1.228 kPa *G*_5_ = 1.618 kPa *G*_∞_ = 0.27 kPa	*β*_1_ = 100 ms^−1^ *β*_2_ = 4.35 ms^−1^ *β*_3_ = 0.2 ms^−1^ *β*_4_ = 0.0053 ms^−1^ *β*_5_ = 5.1 × 10^−6^ ms^−1^
CSF	*ρ* = 1000 kg/m^3^ *C *= 1484 m/s *Г*_0_ = 0.110 S_1_ = 1.979	*µ* = 8 × 10^−4^ Pa·s	

### The Combat Helmet Shell and Pad Models

The FE model of the combat helmet shell (Fig. 1b) is based on the geometry of the advanced Combat Helmet (ACH), which is currently used by the U.S Army.^17^ The model includes 36,510 solid elements. The helmet pad model consists of 62,635 elements, which were assigned into two parts: one-layer of hard foam and one-layer of soft foam, each approximately 10mm thick. There is no gap between the pad and the helmet shell.

The ACH shell is manufactured with woven Kevlar fabric laminate embedded in a thermoset resin. We modelled the shell as an orthotropic material, which allows modelling the material in three orthogonal directions. We defined the material coordinate system to ensure that the helmet shell’s normal direction is aligned with the thickness direction of the composite matrix. As shown in Fig. 1b, each element of the helmet shell has its own local material axes. The material properties for the helmet shell were obtained from Refs. 44, 50 shown in Table 2.

**Table 2:** Material properties of ACH shell.^50^

Density	1230 kg/m^3^	Shear modulus G_13_	2.72 GPa
Young’s modulus *E*_11_	18.5 GPa	Shear modulus G_23_	2.72 GPa
Young’s modulus *E*_22_	18.5 GPa	Poisson’s ratio *ν*_12_	0.25
Young’s modulus *E*_33_	6 GPa	Poisson’s ratio *ν*_13_	0.33
Shear modulus *G*_12_	0.77 GPa	Poisson’s ratio *ν*_23_	0.33

The currently used ACH padding system is Zorbium Action Pad (ZAP^TM^). The padding has a thickness of 20 mm, comprised of one-layer hard and one-layer soft polyurethane foams, with different densities (Table 3). The material behaviour of the polyurethane foams is rate dependent. The Young’s modulus of the hard and soft foams were used as reference modulus along with a decay constant of 5 ms^−1^,^50^ listed in Table 3. The compression behaviour of the two foams were defined with nominal stress-strain curves (tested at strain rate of 200 s^−1^), taken from Ref. 50.

**Table 3:** Material properties of ACH pads.^50^

	Density (kg/m^3^)	Young’s modulus (MPa)
Soft foam	61	0.84
Hard foam	63	8.4

To ensure the helmet pads fit on the head properly, we followed the fitting method in previous studies^19,42^ and conducted a preliminary simulation where the helmet was fitted onto the human head. We defined the skin and helmet shell as rigid body and used the hard and soft foam material properties to model the helmet pads. Then, helmet was moved slowly onto the skin until the pads were slightly deformed and filled the gap between the pads and skin. This process ensured that the helmet was properly fitted onto the head model and no penetration exists between the pads and skin. The coordinates of the pad nodes were exported and used in the following simulations. As only the coordinates of the pad nodes were changed, there will not be any residual stress in the pads.

### The Goggles Model

The FE model of the goggles was based on the standard-issued goggles, shown in Fig. 1c. The geometry and dimensions of the goggles were chosen to fit securely on the skin. The model includes a lens and a liner. The strap and other accessories were ignored as they were assumed to have negligible effect during the short time (a few millisecond) blast exposure. The model consists of 28,228 solid elements and its material properties, reported in Table 4, were taken from Ref. 38. The goggles were fitted onto the skin in a simulation similar to that used to fit the helmet.

**Table 4:** Material properties of goggles components.

Component	Material	Density (kg/m^3^)	Young’s modulus (MPa)	Poisson’s ratio
Lens	Polycarbonate	1220	2400	0.37
Liner	Soft foam	136	1	0.21

### Blast Waves Modelling

**Figure 2:**
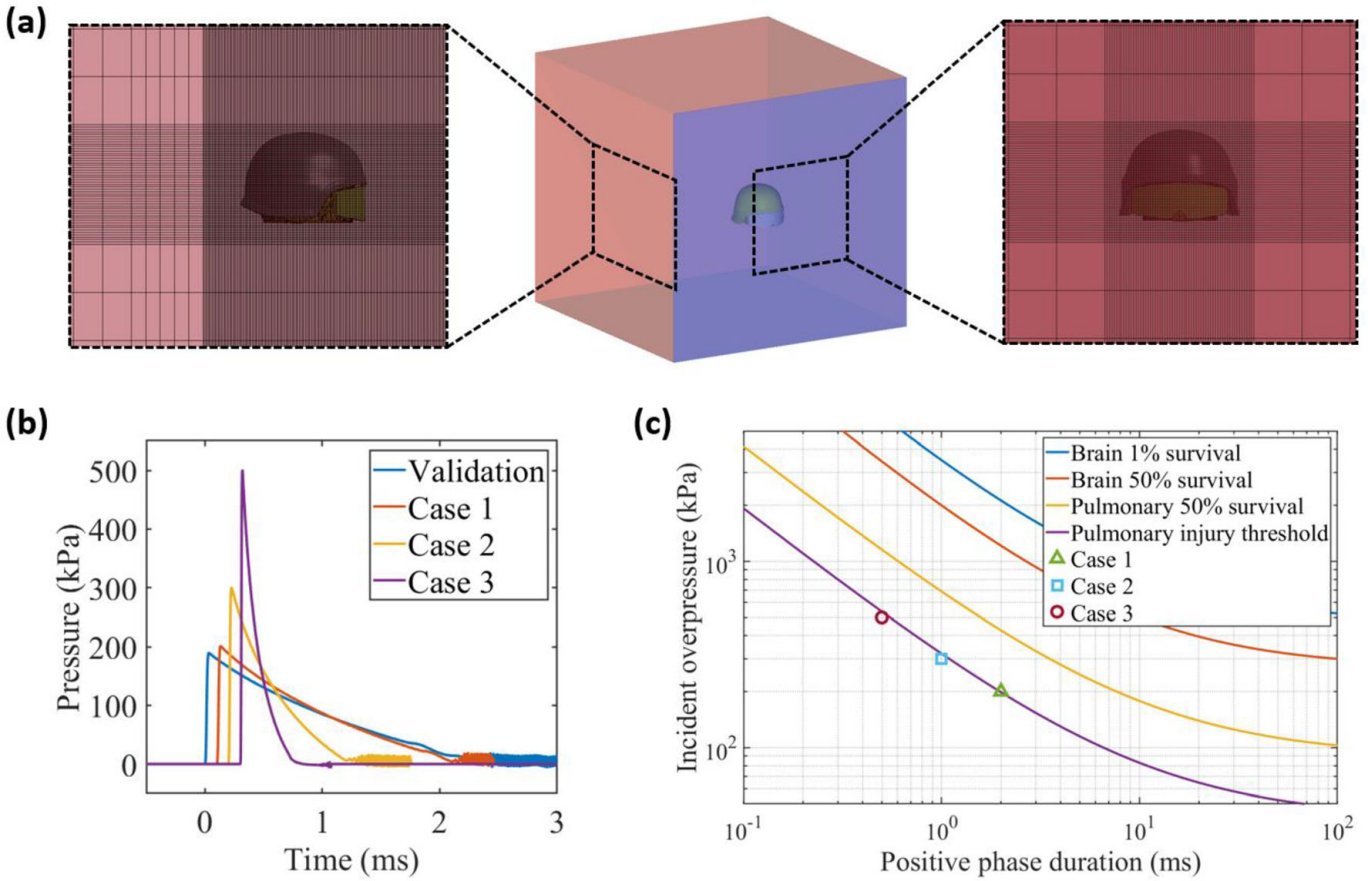
(a) Multi-size air mesh domain. (b) The blast wave pressure histories used for model validation and blast cases. (c) The studied blast cases and brain/pulmonary injury criteria.^5,30^

We used the prescribed inflow and ALE (Arbitrary Lagrangian–Eulerian) methods to generate the blast wave and model the blast wave/head interaction. A multi-size air mesh domain was used to save computational cost and avoid the blast wave reflected from the air domain boundaries reaching the head (Fig. 2a). The air domain includes coarse mesh and refined mesh regions. To ensure a reasonable wave/head interaction, the head model was placed in the refined mesh region, which was meshed with 2.5 × 5 × 5 mm^3^ elements (Fig. 2a). In this refined mesh region, the air mesh size in the blast wave transmission direction was determined to be 2.5 mm, as suggested in Refs. 28,48 The size of the air domain is 1310 × 1250 × 1315 mm and it contains 928,800 hexahedral elements. The mesh convergency study and modelling details can be found in our previous studies.^47,48^ The helmeted head FE model was unconstrained and free to move within the air domain. The time step was determined automatically in LS-Dyna using the acoustic speed and the element lengths.^21^

### Simulated Blast Loadings and Directions

We modelled four blast loadings, including one for validating the FE model and three for studying the brain response, as shown in Fig. 2b and Table 5. The blast loading used for model validation is based on the experiments reported in Ref. 18. Li *et al*.^18^ conducted frontal air blast experiments on a bare dummy head and a helmeted dummy head. They measured the pressures on the surface of the head at several locations, including front, top, rear, side, eye, and ear. We used the reported blast loading to simulate a frontal blast on both the bare head model and the helmet-head model. We validated our head and helmet FE model by comparing the computational predicted pressures and the experimental measured pressures. To study the brain response to blast exposure at different levels, we selected three blast loadings with different incident overpressures and positive phase durations, which are below the pulmonary injury and brain injury thresholds (Fig. 2c).^5,30^

**Table 5:** The parameters of the blast loadings, head orientation and model configurations.

Blast loadings	Peak overpressure (kPa)	Positive phase duration (ms)	Direction	Model configurations
Validation	188.5	2.34	Frontal	Bare head, helmet-head
Case 1	200	2	Frontal, lateral, rear	Bare head, helmet-head, helmet-goggles-head
Case 2	300	1	Frontal, lateral, rear	Bare head, helmet-head, helmet-goggles-head
Case 3	500	0.5	Frontal, lateral, rear	Bare head, helmet-head, helmet-goggles-head

Due to the asymmetric geometries, the head response to blast and the protection performance of helmet and goggles are different under blasts from different directions. Therefore, we investigate three typical blast directions: frontal (anterior-posterior), lateral (left-right) and rear (posterior-anterior). A total of 27 simulations were conducted, including three blast loadings, three blast directions and three model configurations (Table 5).

### Data processing

**Figure 3:**
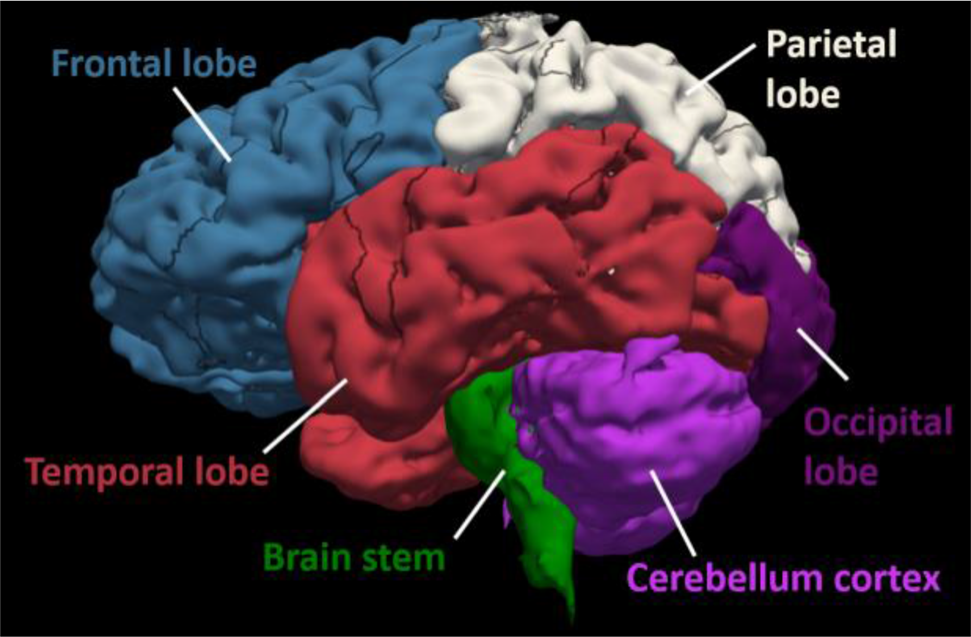
Brain anatomy, including four lobes, cerebellum cortex and brain stem.

The simulation time was set to 2.2 ms as this is enough for the blast wave to interact with the head and helmet/goggles models. Our simulations showed that at 1.6 ms, all brain and CSF elements had already experienced their peak pressures. During the simulation, the data of the brain and CSF/ventricle elements was written into a binary history file. These data were processed to determine the peak positive/negative pressure, strain and strain rate of each element over the entire simulation. The outputs were written in MRI NIfTI format, which allowed us to use FSL, an MRI-based neuroimaging tool, to analyse the data. We created masks of four lobes (frontal, parietal, temporal and occipital), cerebellum cortex and brain stem (Fig. 3), which were used to determine the mean and standard deviation of the negative/positive pressures, strain and strain rate in these regions of interest.

## Results

### Validation of the FE Models

**Figure 4:**
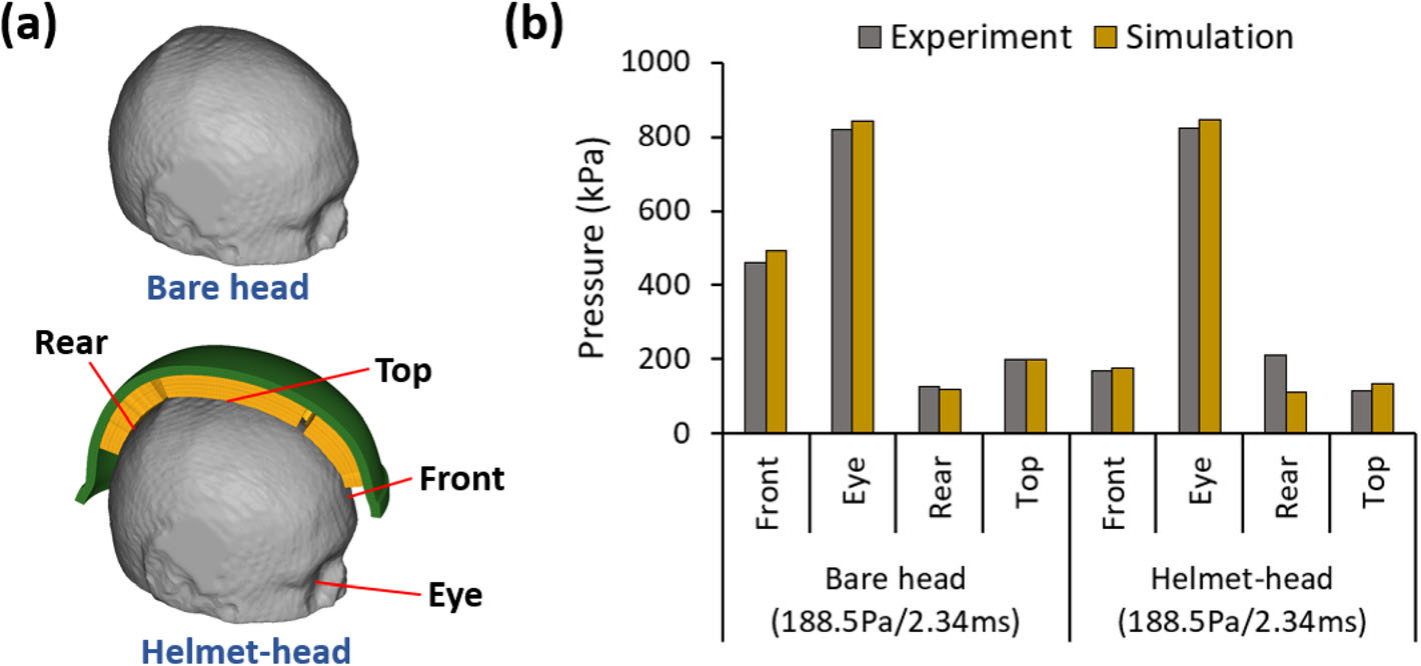
(a) The locations of measured pressures (half of the helmet shell and pads are masked). (b) Comparison of peak pressures predicted by simulation and measured in experiments.^18^

Figure 4a shows the locations where the pressure were measured in the blast experiments^18^ and Fig. 4b compares the recorded and predicted peak pressures for two configurations: bare head and helmet-head. Overall, the peak pressures predicted by simulations are in good agreement with those measured in the experiments. More specifically, in the bare head configuration, the peak pressure differences in all locations are less than 6.2%. In helmet-head configuration, the peak pressure differences at the front (4.5%), eye (2.4%) and top (14.8%) locations are also low. At the rear location, the helmet-head experiment measured 210kPa peak pressure, which is higher than that from simulation (110kPa). This is because of the different geometries of the helmet pads used in the FE model and the experiments. In the FE model, the corresponding rear location is covered by the helmet pad, which mitigates the pressure wave. However, in the helmet-head experiment, there was a gap between the helmet shell and pads, which led to the underwash effect.^24,34^ The underwash effect results from the blast wave collision within the gap, creating a pressure spike.

### Brain Biomechanical Response to Frontal Blast

#### Intracranial Pressure

**Figure 5:**
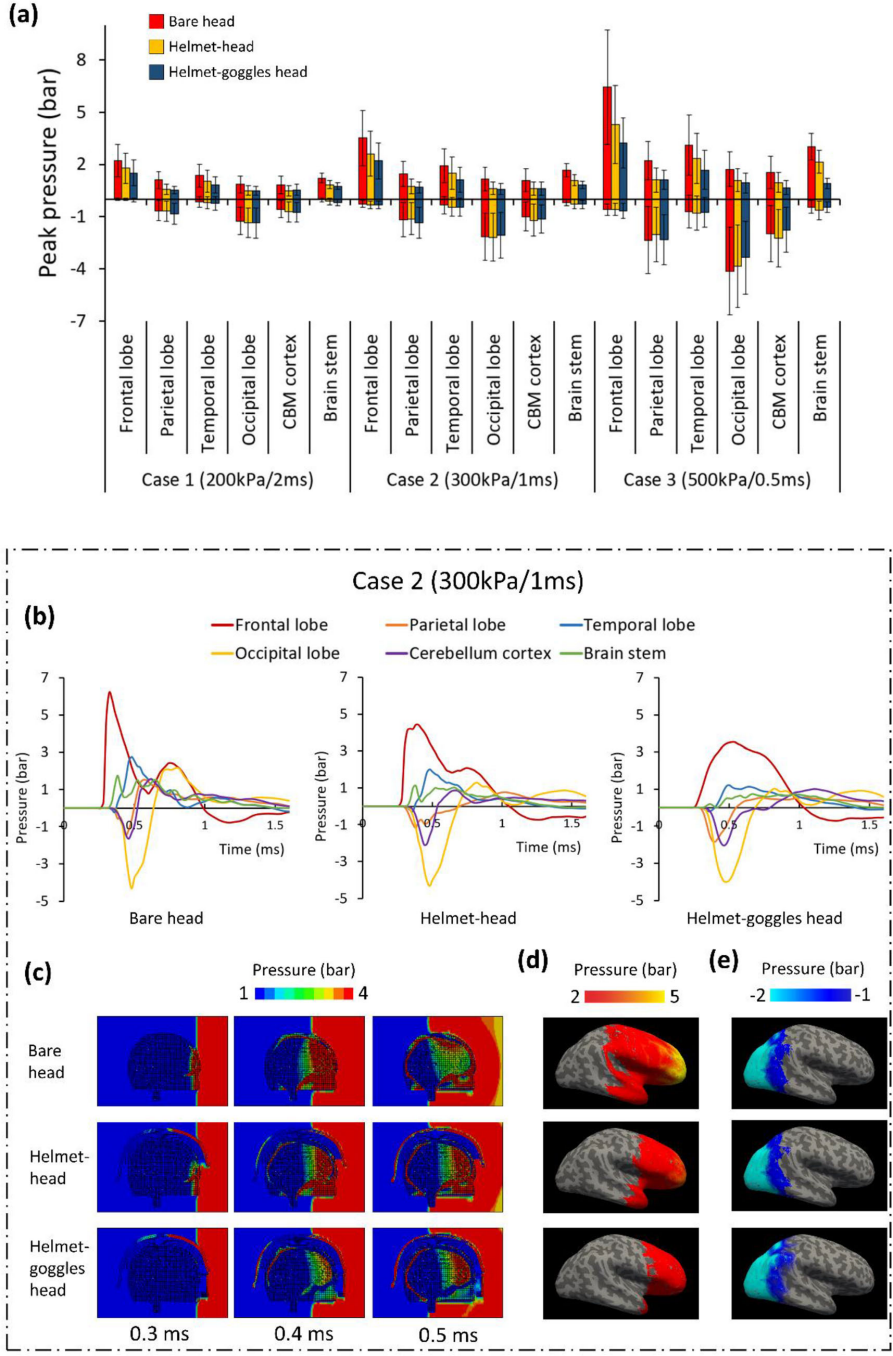
Frontal blast results: (a) Comparison of peak positive and negative ICP in six regions (CBM: cerebellum). (b) Pressure time histories measured at the six regions. (c) Pressure contours across the head in the midsagittal plane. (d and e) The peak positive and negative ICP measured at the middle layer of cortex and projected onto an inflated brain image (light grey: gyral regions and dark grey: sulcal regions).

We determined the mean peak positive and negative ICPs in six regions (four lobes, cerebellum cortex and brain stem) (Fig. 5a). For all blast loadings, the positive ICP was lowest in the helmet-goggles-head model followed by the helmet-head model. Wearing a helmet reduced the positive ICP by 34.9% (Case 1), 37.2% (Case 2) and 35.1% (Case 3) while wearing both helmet and goggles led to 40.8% (Case 1), 46.9% (Case 2) and 53.0% (Case 3) reductions. In each blast case, the frontal lobe experienced the highest positive ICP, then the temporal lobe. The cerebellum cortex and occipital lobe experienced relatively lower positive ICPs.

Regarding the mean negative ICP, the contrecoup regions (occipital lobe, cerebellum cortex and parietal lobe) experienced large negative pressures, with largest values observed in the occipital lobe (Fig. 5a). Helmet and goggles did not have a consistent effect on the negative ICP. In some regions and load cases, helmet increased the negative ICP while in others, helmet had the opposite effect. Overall, among the three models, changes in negative ICP were smaller than changes in the positive ICP.

 Figure 5b shows the pressure time histories predicted by the three models at the six regions for Case 2. The presence of the helmet significantly reduced the positive pressures in all regions, particularly in the coup region, such as the frontal and temporal lobes. Adding the goggles led to a further reduction in the positive pressure. Moreover, the rising edge of the pressure in the frontal lobe was also weakened by the goggles. In contrast, in the contrecoup region, such as the occipital lobe, parietal lobe and the cerebellum cortex, the helmet and goggles did not show notable effects on the negative ICP.

Figure 5c illustrates the pressure contours at different times for the three models under load case 2. At 0.3 ms, the blast wave has already reached the head in the bare head model. The bare head is directly exposed to the blast wave, bearing larger ICP. At this time, the helmet impeded the blast wave at the upper head area, mitigating some pressure wave transmission into the head. The blast wave is further impeded by the goggles in the helmet-goggles head model. These protective effects are further demonstrated at 0.4 and 0.5 ms. Notably, the blast wave initiated a pressure wave in the helmet shell, which transmits faster than pressure waves in the head, due to its higher acoustic impedance.

To assess how helmet and goggles affected the distribution of ICP, we projected the peak positive and negative ICP measured at the middle layer of the cortex onto an inflated image of the brain (Figs. 5d and 5e). Again, the contours show that the positive ICP at the coup region has been reduced both in magnitude and area. However, minimal changes are seen in the negative ICP at the contrecoup region. Additionally, there is no obvious difference between the ICP contours in the sulci and gyri regions, suggesting the anatomical features of the brain does not affect brain response to ICP.

#### CSF Cavitation

**Figure 6:**
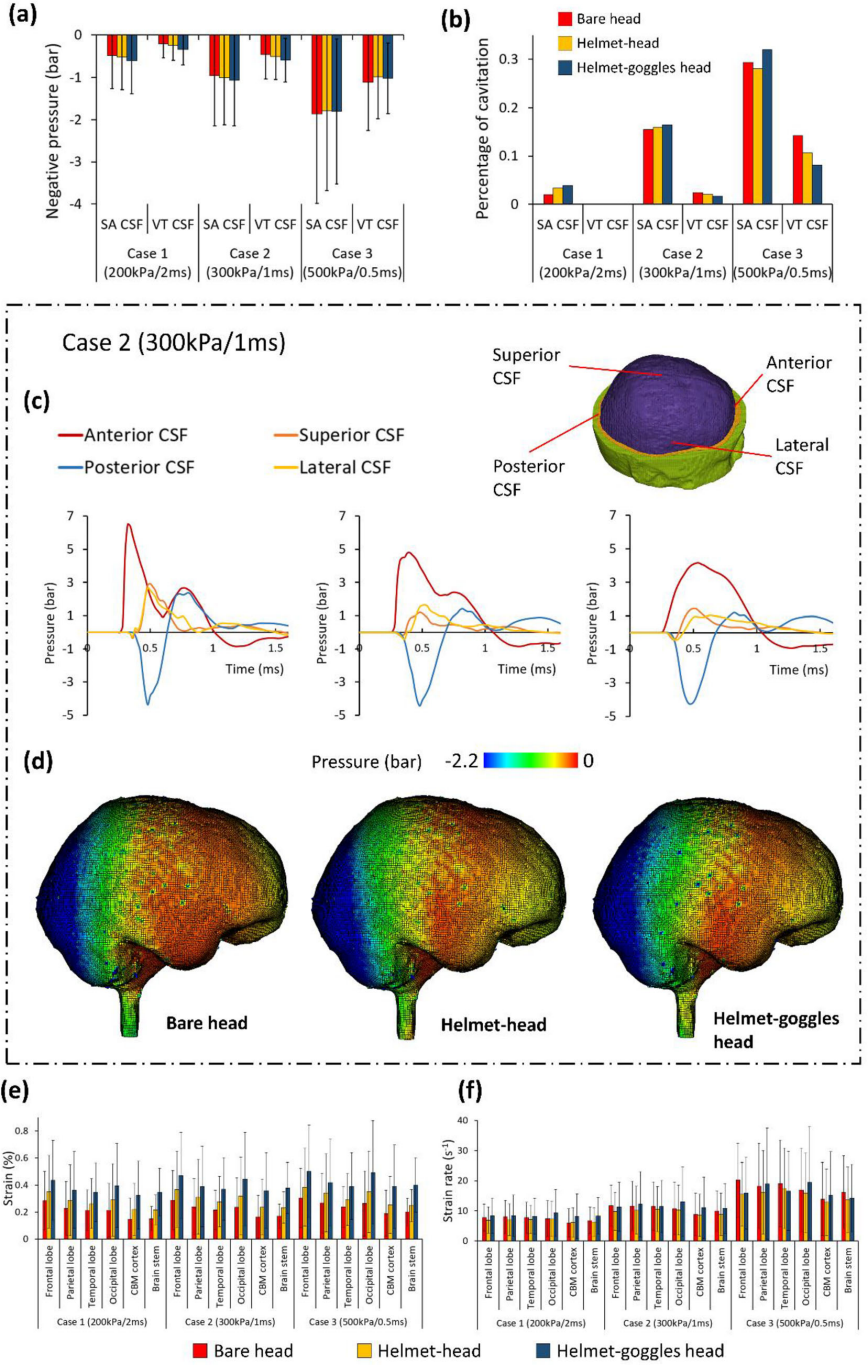
Frontal blast results: (a) The mean negative pressure of the CSF and (b) the percentage of CSF cavitation (*SA* subarachnoid; *VT* ventricular). (c) Pressure time histories measured at four locations of the subarachnoid CSF. (d) The peak negative pressure contours of CSF. (e, f) Mean value of the peak maximum principal strain and strain rate (*CBM* cerebellum).

We firstly plotted the mean negative pressure measured at the entire subarachnoid CSF and ventricular CSF (Fig. 6a). Then, we determined the cavitation severity by calculating the percentage of CSF elements that experienced negative pressure lower than the cavitation pressure threshold, - 2.2bar, a conservative value based on previous experimental studies.^6,46^ Figures 6a and 6b show that the helmet and goggles did not have consistent effects on both the CSF pressure and cavitation percentage. In Case 1 and 2, the CSF cavitation was increased by the presence of helmet and goggles. However, in Case 3, the opposite effect was observed. Although the cavitation percentages of ventricular CSF in Case 3 had slightly larger differences, the ventricular CSF only accounts for 6% of the total CSF. Therefore, our results show that the overall effect of helmet and goggles on mitigating CSF cavitation is minimal.

We performed further analysis on load case 2. Figure 6c shows the pressure histories measured at four locations across the subarachnoid CSF. The shapes and magnitudes of the pressure histories at the anterior and posterior CSF are quite similar with those measured at the frontal and occipital lobes, due to their similar positions relative to the blast wave. The effects of the helmet and goggles on the CSF pressure are also similar with those on the ICP. The positive pressure at the coup region (anterior CSF) was mitigated significantly by the helmet and goggles but the negative pressure at the contrecoup region (posterior CSF) was changed marginally. These results can be further supported through the peak negative pressure contours of CSF, shown in Fig. 6d. These contours show that the helmet and goggles did not have a noticeable effect on changing the distribution of the negative pressure across the CSF.

#### Strain and Strain Rate

Figures 6e and 6f compares the mean value of the peak maximum principal strain and strain rate in the six regions of interest for the three blast cases. In the bare head model, the mean strain ranged from 0.15 to 0.3%. The range increased to 0.22–0.35% with the helmet and further increased to 0.32–0.5% with both helmet and goggles. On average, wearing a helmet increased the brain strain by 32% while wearing both helmet and goggles increased the brain strain by 85%. In each case, the frontal lobe sustained the highest strain while the cerebellum cortex sustained the lowest strain. Overall, the strain level remained quite low during the simulation time (2.2 ms).

The mean strain rate in the brain was not affected much by the helmet and goggles. The helmet alone reduced brain strain rate between 6 and 12%. However, the goggles did not have a consistent effect on brain strain rate. Wearing both helmet and goggles may increase or decrease the brain strain rate, with changes between − 3 and 17%. It should be noted that the standard deviation of the strain rate distribution in our regions of interest is very high, with some regions undergoing strain rates of up to 200 s^−1^.

### Brain Biomechanical Response to Lateral Blast Exposure

#### Intracranial Pressure

**Figure 7:**
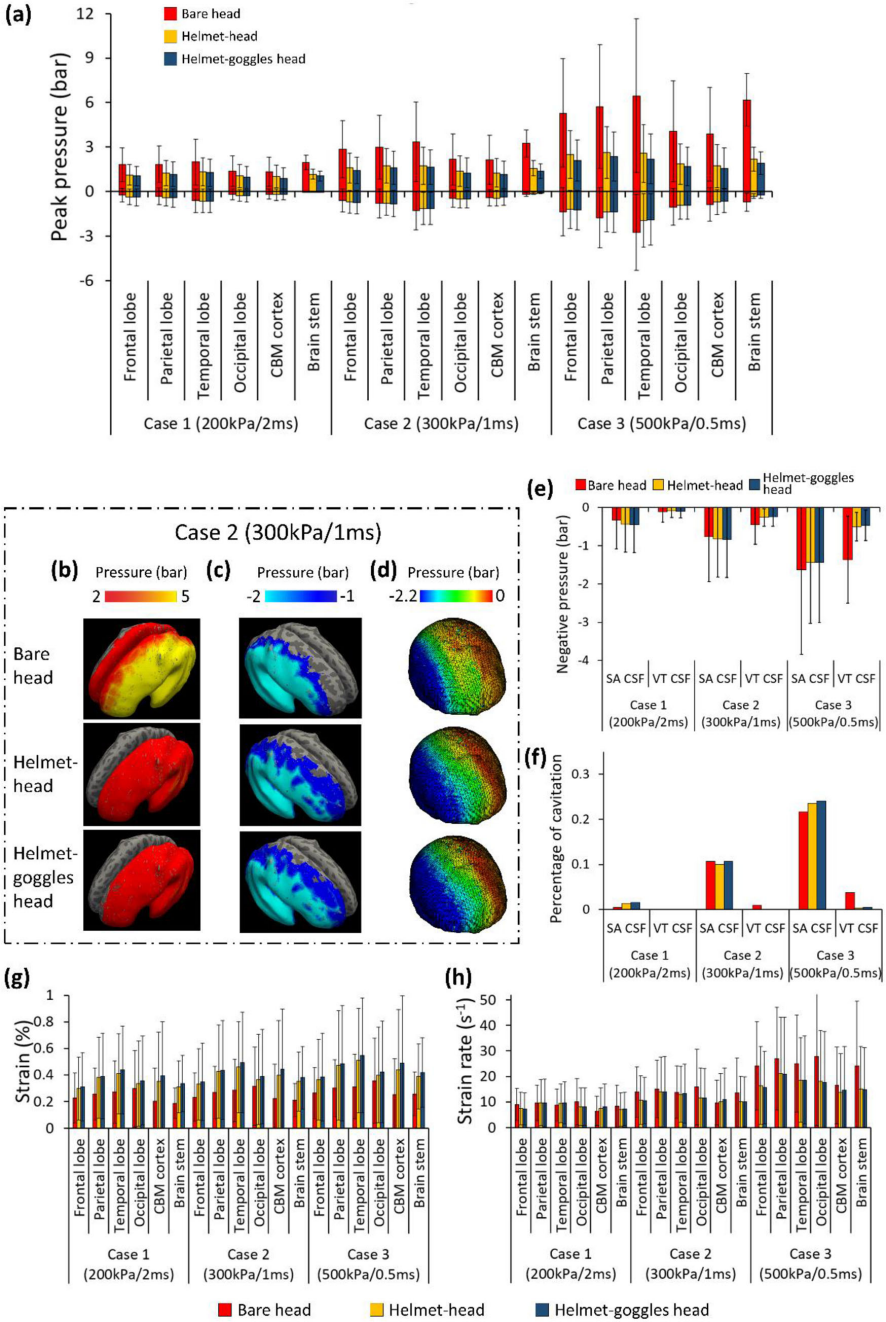
Lateral blast results: (a) Comparison of mean peak positive and negative ICP in the six regions (CBM: cerebellum). (b, c) The peak positive and negative ICP measured at the middle layer of cortex and projected onto an inflated brain image (light grey: gyral regions and dark grey: sulcal regions). (d) The peak negative pressure contours of CSF. (e) The mean negative pressure of the CSF (SA: subarachnoid; VT: ventricular). (f) The percentage of CSF cavitation. (g, h) Mean value of the peak maximum principal strain and strain rate (CBM: cerebellum).

Figure 7a shows the comparison of the mean peak positive and negative ICPs in six regions of interest under lateral blast. On average, wearing a helmet reduced the positive ICP by 32% (Case 1), 43.9% (Case 2) and 57% (Case 3). Wearing both helmet and goggles reduced the positive ICP by 37.6% (Case 1), 48.9% (Case 2) and 62% (Case 3). In all blast cases, the temporal lobe experienced the highest positive ICP. The helmet and goggles did not have a significant effect on the negative ICP in Case 1 and Case 2. In Case 3, the helmet and helmet-goggles reduced the negative ICP by 24.8 and 27.6% on average.

Figures 7b and 7c shows the peak positive and negative ICP measured at the middle layer of the cortex in Case 2, projected onto an inflated image of the brain. The left hemisphere (coup) experienced large positive ICP while the right brain experienced large negative ICP. When protected by the helmet and goggles, the positive ICP within the brain was reduced significantly. However, only small changes were found in the negative ICP distribution at the right hemisphere. In addition, no obvious difference was found in the ICP distributions between sulci and gyri regions.

#### CSF Cavitation

Figures 7e and 7f summarizes the mean negative pressure and percentage of cavitation in the subarachnoid CSF and ventricular CSF. In load case 1, the mean negative pressure in all configurations were low (up to − 0.5 bar) and the percentage of cavitation was also low in both subarachnoid and ventricular CSF (less than 1.5%). In all cases, percentage of cavitation in ventricular CSF was small. However, percentage of cavitation in subarachnoid CSF was high under load cases 2 and 3 (over 10 and 20% respectively). Wearing the helmet and goggles had a marginal effect on the mean negative pressure in CSF and the percentage of cavitation. This conclusion is further supported by the peak negative pressure contours of CSF shown in Fig. 7d, which shows similar negative pressure distributions.

#### Brain Strain and Strain Rate

Figure 7g shows the mean value of the peak maximum principal strain in the brain. In the bare head model, the mean strain ranged from 0.19 to 0.36%. Wearing a helmet increased this range to 0.30 to 0.51% while wearing both helmet and goggles increased it to 0.31 to 0.55%. On average, wearing a helmet increased the brain strain by 50% while wearing both helmet and goggles increased the brain strain by 61%. In each case, the temporal lobe sustained the highest strain. Figure 7h compares the mean value of strain rate within the brain. Overall, the helmet alone reduced brain strain rate between 5 and 28%. However, the goggles did not have a consistent effect on brain strain rate. Similar with the frontal blast results, the standard deviation of the brain strain rate is high, indicating that some brain regions undergo relatively large strain rates.

### Brain Biomechanical Response to Rear Blast Exposure

#### Intracranial Pressure

**Figure 8:**
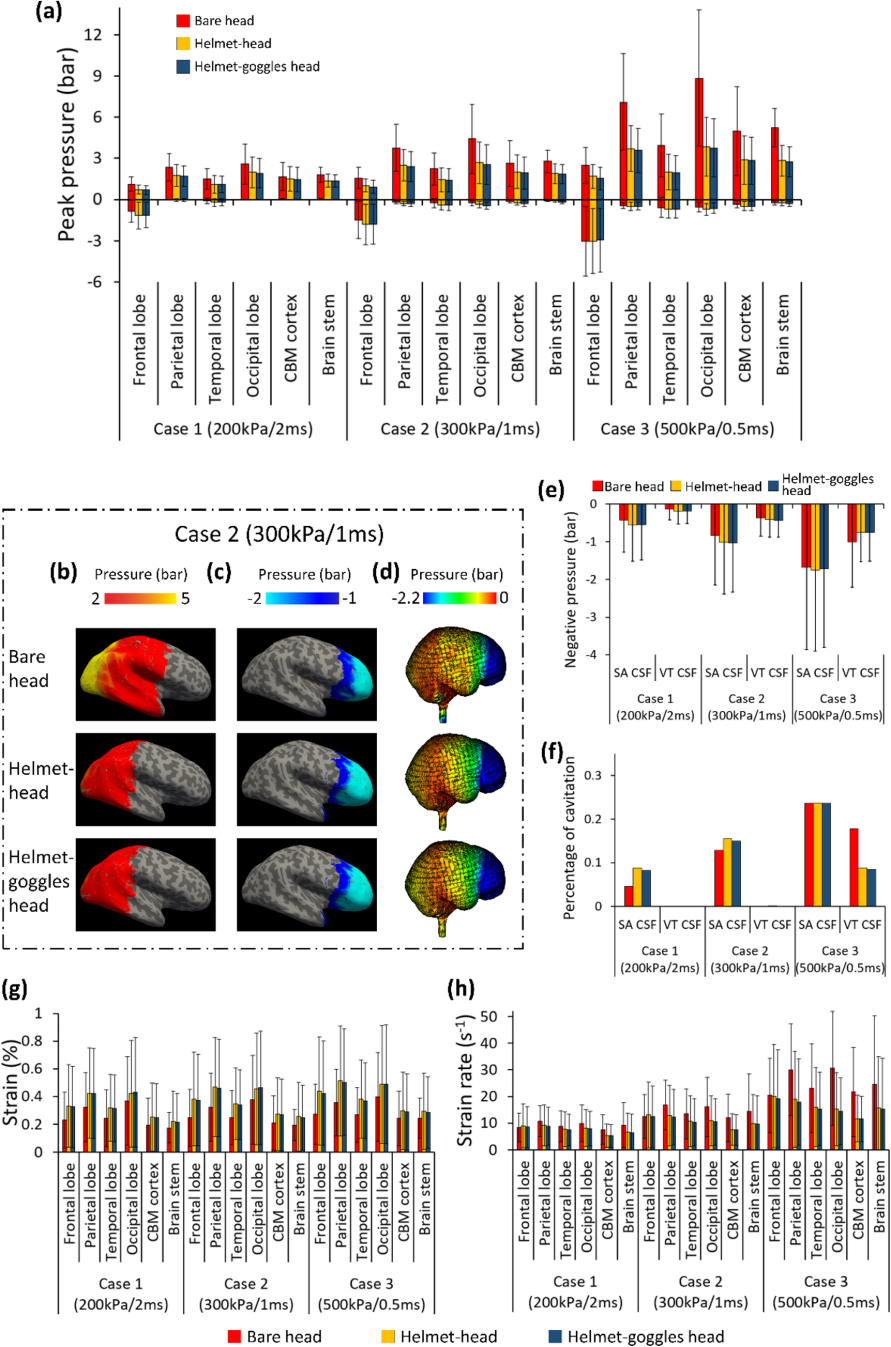
Rear blast results: (a) Comparison of mean peak positive and negative ICP in the six regions in all cases (CBM: cerebellum). (b, c) The peak positive and negative ICP measured at the middle layer of cortex and projected onto an inflated brain image (light grey: gyral regions and dark grey: sulcal regions). (d) The peak negative pressure contours of CSF. (e) The mean negative pressure of the CSF (*SA* subarachnoid; *VT* ventricular). (f) The percentage of CSF cavitation. (g, h) Mean value of the peak maximum principal strain and strain rate (*CBM* cerebellum).

Figure 8a shows the mean peak positive and negative ICP in the regions of interest in all blast cases. Wearing the helmet reduced the positive ICP by 24.1% (Case 1), 33.2% (Case 2) and 45.7% (Case 3) on average. Wearing goggles and helmet led to 26.1% (Case 1), 36% (Case 2) and 47.8% (Case 3) reduction in the positive ICP. Therefore, the goggles reduced the positive ICP by less than 3%. In terms of negative ICP, the helmet and googles had negligible effects.

The peak positive and negative ICP measured at the middle layer of the cortex in Case 2 was projected onto an inflated image of the brain, as shown in Figs. 8b and 8c. Similar with the frontal blast exposure results, the coup region experienced large positive ICP in bare head configuration, and the pressure was mitigated notably by the helmet but not the goggles. For negative ICP distributions, no obvious difference was observed when wearing the helmet and goggles. Again, no obvious difference was found in the ICP distributions between sulcal and gyral regions.

#### CSF Cavitation

The mean negative pressure and percentage of cavitation in CSF were increased with the increase in blast intensity (Figs. 8e and 8f). The helmet and goggles did not have large effects on the CSF cavitation and these effects are not consistent either. Figure 8d shows that the helmet and goggles do not have a noticeable effect on the peak negative pressure contours of CSF in the rear blast. These contours are similar to those in frontal blast cases, where contrecoup regions experienced cavitation.

#### Brain Strain and Strain Rate

As shown in Fig. 8g, the brain strain in bare head model ranged from 0.17 to 0.40%, which was increased to 0.22–0.47% when wearing the helmet and 0.25–0.50% when wearing the helmet and goggles. The average increase in strain was 34% (helmet) and 32% (helmet and goggles). This suggests that the goggles slightly decreased the average strain in the brain in rear blast exposure. The coup regions, parietal and occipital lobes had the highest strain. Figure 8h compares the mean value of strain rate within the brain. Overall, the helmet alone reduced brain strain rate between 16 and 34%. However, the goggles had a small effect on brain strain rate. Again, the standard deviation of the brain strain rate was high, suggesting that high strain rate was experienced at some regions.

### Head Kinetic Energy in All Loading Conditions

**Figure 9:**
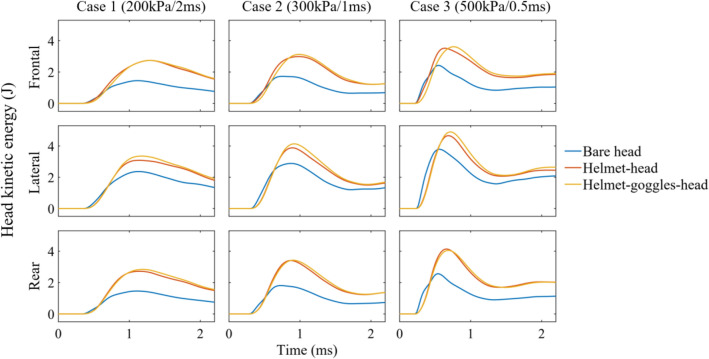
Head kinetic energy of the three model configurations in all loading conditions.

Next, we determined the head kinetic energy time histories for the three model configurations and all the loading cases and directions, as shown in Fig. 9. For each loading condition, head kinetic energy of the bare head model is smaller than those in the helmet-head and helmet-goggles-head models. Compared with helmet-head model, the head kinetic energies of helmet-goggles-head model are slightly higher in most loading conditions. These suggest that the presence of helmet significantly increase the head kinetic energy, resulting from the increased impulse due to the increased area subjected to blast wave. The effect of goggles on head kinetic energy is smaller than the helmet. These explain why the brain strain was larger in the models with helmet and goggles.

## Discussion

This study comprehensively assessed the protective capability of the advanced combat helmet and goggles against primary blast exposure by comparing various biomechanical responses of the brain to three blast loading conditions and directions. In addition to the ICP, brain strain and strain rate, we for the first time studied the effects of wearing the helmet and goggles on mitigating CSF cavitation.

The helmet is found to be very effective in reducing the positive ICP in all blast loadings and directions (24–57% reduction). This reduction is more profound in high-intensity blast cases. However, wearing the helmet did not have a consistent effect on the negative ICP and the effects were marginal. These findings agree with previous experimental and computational studies.^25,50^ Interestingly, we found that the helmet has an adverse effect on CSF cavitation: it slightly increased the negative pressure and the percentage of cavitation in CSF. In our recent study,^49^ we experimentally observed the CSF cavitation in a simplified head surrogate under blast wave. Using FE simulation, we found that the blast wave initiates two pressure waves in the surrogate model: the outer wave propagating in the skull surrogate and the inner wave propagating across the brain and CSF. Due to a higher speed, the outer wave reaches the contrecoup skull first and initiates a tensile wave at the CSF-brain interface, inducing cavitation bubbles in the contrecoup CSF. Shortly, the inner wave reaches the contrecoup zone and collapses the cavitation bubbles. The helmet’s adverse effect on CSF cavitation suggests that the helmet may increase the blast wave transmission into the head, which is likely due to the helmet pads. Singh *et al*. compared the performance of soft pads and strap suspension and found that strap suspension was more effective in mitigating blast wave than pads.^36^ This conclusion was further supported by Opteynde et al.^26^ where they found that historical helmets, which used strap suspension, had similar or better blast wave mitigation performance than modern helmets with pad liners. In contrast to strap suspension where the helmet shell is separated from the head, the pads deform and fit the head, creating a pathway for the pressure wave to transmit from the helmet shell to head. Foam materials have the capability to mitigate pressure wave. However, the mitigation performance highly depends on their thickness. The experiments in Ref. 29 showed that when exposed to a blast wave from 170g-C4 charge, a 20mm-thick foam is not able to mitigate the pressure wave while a 60mm-thick foam can significantly reduce the pressure. As the thickness of pads currently used in the advanced combat helmets is only 20 mm, the pads probably cannot mitigate the pressure waves transmitted into the head and the subsequent CSF cavitation. Considering that helmet increased the area subjected to blast wave, these factors may direct more blast wave into the head and increase CSF cavitation.

Wearing goggles had a minimal effect on the positive ICP in the rear blast, but it was effective in frontal and lateral blast exposures. Goggles introduce a large gap filled with air between themselves and the head, impeding the blast wave from interacting with the head directly. Goggles act similar to a face shield or a visor. Several studies^31,33,36,39,43^ have shown the excellent ability of face shields in mitigating the ICP. For example, Tse *et al*.^43^ studied the protective performance of single-layered and multi-layered face shields for frontal blast exposure. They showed that the multi-layered face shield provides a better blast wave attenuation performance, as it benefits from the polycarbonate exterior shell for structural integrity and inner aerogel filler for blast wave mitigation. However, the face shield adds additional weight to the helmet and restricts movement and vision. Previous work has shown goggles’ ability in mitigating eye injury.^2,38^ Therefore, goggles may be a more practical solution, providing protection to both brain and eyes. Goggles’ effect on mitigating CSF cavitation was not consistent across the blast loads and directions, and its overall protection effects was marginal. It is likely that the CSF cavitation is mainly induced by the blast wave transmitting from the helmet to the head. Therefore, wearing goggles or a face shield probably will not mitigate CSF cavitation.

Our results showed that helmet and goggles increased strain across the brain in all blast cases and head orientations. This conclusion is different to that in a previous study,^50^ where the authors found helmet could reduce brain strain by 30% in average. The reason for the different conclusions is not clear. However, the comparison of head kinetic energy suggests that wearing helmet and goggles increased the area subjected to the blast wave, which increased the impulse applied on the head and further increased the head kinetic energy. Under primary blast wave, brain’s volumetric deformation is small, and a large portion of the strain is produced by shear deformation, which is mostly determined by the head motion. Here, we only simulated 2.2 ms of the blast exposure, where the head motion is small. The predicted brain strain in all loading scenarios is less than 1%. However, after blast exposure, the head may experience large motion, particularly with the engagement of neck. Such large head motion can induce large strains across the brain, which suggests that the adverse effect of the helmet and goggles may be more profound after blast exposure. For strain rate, the helmet shows consistent mitigating effects while goggles’ effect on strain rate is small and inconsistent.

The effects of the helmet and goggles were more profound in high-intensity blast cases. Besides, head orientation affects the brain responses and the protective effects of helmet and goggles. However, similar with previous studies,^15,50^ the general distributions of brain responses are similar among the three head orientations: at coup regions, brain and CSF undergo positive pressures and at the contrecoup regions, they undergo negative pressures. In addition the pressure distributions in the brain are not affected by the anatomical features (sulci and gyri), which is different to those in impact induced brain responses.^12^

Currently, the most commonly used blast injury thresholds are based on the parameters of blast waves: peak overpressure and positive phase duration. ICP has been measured in many studies. However, the link between ICP and brain injury is unclear. The strain we measured in this study is the first principal strain, which is between 0.4 and 0.6%. This strain level is far from the strain threshold, determined in previous studies, which suggested brain injury occurs with at least 13% strain.^3,9,16,23^ In the present simulations, the brain’s volumetric deformation is limited as the bulk modulus of the brain tissue around 2.2 GPa. Therefore, for example, under 6 bar ICP, the volumetric strain is only 0.03%. In fact, the measured strain is mainly the shear strain of the brain. However, the brain’s shear strain is primarily determined by the kinematics of the head. During the short-time (less than 2.2 ms) of primary blast wave exposure, the brain’s shear deformation is limited. This is different to impact induced TBI, where impact loadings interact with head for 10–20 ms and the strain can reach 50%.^12^ However, some cortical regions underwent strain rates as high as 200 s^-1^, which is larger than some strain rate thresholds for brain injury suggested in previous studies. According to,^4^ neuronal damage occurs at strain rates between 10 and 75 s^−1^. Recently, a study investigated *in vitro* brain tissue response to blast waves and suggested 25 to 33 s^−1^ strain rates as a threshold for deficits in long-term potentiation.^45^ These indicate that strain rate is likely to be a mechanism for producing blast TBI.

Our results suggest that in most blast loads and directions studied here, the helmet slightly increased the risk of CSF cavitation by increasing both the negative pressure in CSF and the percentage of cavitation in CSF. Here we used − 2.2 bar as the CSF cavitation threshold, as determined in our previous work.^46^ This value is slightly more conservative than the -1.8bar threshold determined by Bustamante *et al*.^6^ Our results showed that 2% of the subarachnoid CSF experienced cavitation under the 200 kPa blast while this percentage climbed to 29.4% under the 500 kPa blast. Our previous study found that CSF cavitation severity is mainly linked to the peak incident overpressure rather than the positive phase duration.^46^ Our results agree with previous experimental study on blast-induced cavitation in post-mortem human head, which found that cavitation occurs from around 140 kPa incident blast.^32^ These results indicate that severe CSF cavitation can occur at blast exposures that are classed as non-lethal.^5,30^ Moreover, our results show that the helmet is likely to increase the severity of CSF cavitation. Future work should include the CSF cavitation risk when assessing brain injury under primary blast exposures and the mitigation effects of protective equipment.

Our study has several limitations. First, the simulation time was set as 2.2 ms, which was a compromise considering size of air domain and computational cost. Although the effects of blast wave can be studied during this short duration, the head motion is minimal, resulting in low strains in brain tissue. As wearing helmet and goggles significantly increased the impulse to the head, the brain strain induced by head motion should be investigated in future work. Secondly, the simulated blast wave only contained the positive pressure phase and ignored the negative pressure phase. The negative pressure phase, usually referred to as blast wind, can cause head oscillations with large rotational accelerations.^13,41^ The effects of the primary blast wave can be studied by using the positive pressure phase alone and future studies may improve our understanding of the effects of the bast wind effect on brain response, particularly brain strain.

In summary, this study presented a comprehensive assessment of the protective performance of the advanced combat helmet and goggles, including their effect on the ICP, CSF cavitation and brain strain and strain rate. It was found that the helmet and goggles reduced the positive ICP while they had either no or adverse effects on CSF cavitation, brain strain and strain rate. For improving blast wave protection, improvement or modification of the helmet shell is difficult as the helmet shell needs to provide primary protection against blunt impact and ballistic loadings. Thus, future work should focus on developing novel helmet pads, e.g., made of hollow structures and multi-layer foams, which are able to reflect and impede blast wave transmission into the head.
